# Lung Cancer Screening in Cancer Survivors vs Those Without a History of Cancer

**DOI:** 10.1001/jamanetworkopen.2025.35000

**Published:** 2025-09-30

**Authors:** M. Patricia Rivera, Thad Benefield, Danielle D. Durham, Lindsay M. Lane, Kevin Fiscella, Supriya Mohile, Louise M. Henderson

**Affiliations:** 1 Department of Medicine, Division of Pulmonary and Critical Care Medicine, The University of Rochester Medical Center, Rochester, New York; 2 Wilmot Cancer Institute, University of Rochester, Rochester, New York; 3 Department of Radiology, The University of North Carolina, Chapel Hill; 4 Department of Family Medicine, The University of Rochester Medical Center, Rochester, New York; 5 Department of Medicine, Division of Hematology and Oncology, The University of Rochester Medical Center, Rochester, New York; 6 Lineberger Comprehensive Cancer Center, The University of North Carolina, Chapel Hill

## Abstract

**Importance:**

Cancer survivorship in the US is increasing greatly, and cancer survivors are at increased risk of developing second primary cancers, with lung cancer being the most common. However, data on lung cancer screening (LCS) with low-dose computed tomography (LDCT) in cancer survivors are lacking.

**Objective:**

To compare characteristics, LDCT interpretation, lung cancer detection rate (CDR), and all-cause mortality rate among cancer survivors vs individuals without prior cancer undergoing LCS.

**Design, Setting, and Participants:**

This cohort study used North Carolina Lung Screening Registry (NCLSR) data from 2015 to 2019 among individuals undergoing LCS at 8 sites in North Carolina. The NCLSR was linked with the North Carolina Central Cancer Registry from 2000 to 2020 to identify cancer survivors and individuals without prior cancer. Data analysis was performed from June 2024 to April 2025.

**Exposure:**

Undergoing LCS.

**Main Outcomes and Measures:**

The primary outcome was whether lung cancer was diagnosed within 1 year after the first LCS examination. Comparison of demographic characteristics between cancer survivors and those without prior cancer was performed with χ^2^ tests. The association of previous cancer with LDCT interpretation, lung CDR, and all-cause mortality rate was modeled using logistic regression.

**Results:**

Of a total of 7295 individuals (mean [SD] age, 64.71 [6.34] years), 814 were cancer survivors (425 men [52.2%]), and 6481 (3290 men [50.8%]) had no history of cancer. Compared with the individuals without prior cancer, cancer survivors were older (3267 individuals without prior cancer [50.4%] vs 501 cancer survivors [61.6%] were aged ≥65 years; χ^2^_1_ = 35.93; *P* < .001), were more likely to be Black than White or other races (871 individuals without prior cancer [13.4%] vs 137 cancer survivors [16.8%] were Black; χ^2^_2_ = 12.46; *P* = .002), were more likely to have formerly vs currently smoked (2728 individuals without prior cancer [42.7%] vs 418 cancer survivors [51.9%] formerly smoked; χ^2^_2_ = 24.62; *P* < .001), and had higher rates of respiratory comorbidities (1834 individuals without prior cancer [35.0%] vs 268 cancer survivors [39.9%]; χ^2^_1_ = 6.19; *P* = .01) and cardiovascular comorbidities (3043 individuals without prior cancer [58.1%] vs 432 cancer survivors [64.4%]; χ^2^_1_ = 9.70; *P* = .002). Cancer survivors and those without prior cancer had similar adjusted rates of positive LDCT results (120 of 758 cancer survivors [15.8%] vs 1032 of 6059 individuals without prior cancer [17.0%]; χ^2^_1_ = 0.86; *P* = .35). The adjusted lung CDRs were 26.0 cases per 1000 (95% CI, 17.0-38.2 cases per 1000) among cancer survivors vs 17.0 cases per 1000 (95% CI, 14.1-20.6 cases per 1000) among those without prior cancer (χ^2^_1_ = 3.38; *P* = .07); the all-cause mortality rates were 19.4 cases per 1000 (95% CI, 12.0-31.3 cases per 1000) among cancer survivors vs 17.1 cases per 1000 (95% CI, 14.1-20.6 cases per 1000) among those without prior cancer (χ^2^_2_ = 0.25; *P* = .62).

**Conclusions and Relevance:**

In this cohort study of LCS-screened individuals, cancer survivors were more likely to be older and formerly smoked and had slightly more comorbidities compared with individuals without prior cancer. Although rates of positive LDCT findings were similar, these findings suggest that the lung CDR may be higher in cancer survivors vs those without prior cancer and should be evaluated in larger studies. The complex relationship between cancer survivorship and the risk of second primary lung cancer underscores both the challenges and the opportunities for preventive care, such as LCS, in this population.

## Key Points

**Question:**

What are the differences between cancer survivors and individuals without prior cancer undergoing lung cancer screening (LCS)?

**Findings:**

In this cohort study of 814 cancer survivors and 6481 individuals without prior cancer undergoing LCS, cancer survivors were more likely to be older and have increased comorbidities. Rates of positive low-dose computed tomography scans were similar between the 2 groups, but rates of lung cancer detection and all-cause mortality were slightly higher among cancer survivors.

**Meaning:**

These findings suggest that LCS may result in early detection of second primary lung cancer in cancer survivors and should be evaluated in larger studies.

## Teaser

This cohort study compares characteristics, low-dose computed tomography interpretation, lung cancer detection rates, and all-cause mortality rates among cancer survivors vs individuals without prior cancer undergoing lung cancer screening.

## Introduction

With the combination of an increasingly aging (≥65 years) population^1^ and medical advances that have dramatically improved cancer survival rates, cancer survivors are a growing segment of the US population. In 2022, there were 18.1 million estimated cancer survivors, with a projected increase to 26 million by 2040.^2^ It is projected that by 2040, close to 75% of cancer survivors will be aged 65 years and older.^3,4^ Cancer survivorship highlights not only medical successes in the early detection and treatment of cancer but also the unique set of health challenges these individuals face, including the increased risk of developing a second primary cancer (SPC).

A cohort study between 1997 and 2014 of 457 334 Danish adults aged 40 years and older with a prior cancer diagnosis (excluding nonmelanoma skin cancer) found that 12.3% (56 114 individuals) developed an SPC.^5^ The cumulative incidence of an SPC was 6.3% (95% CI, 6.2%-6.4%) at 5 years, 10.5% (95% CI, 10.4%-10.6%) at 10 years, and 13.5% (95% CI, 13.4%-13.7%) at 15 years. Lung cancer was the most common or second most common SPC and was associated with the highest 10-year cumulative incidence.^5^ A systematic analysis of incidence and mortality data from 1992 to 2017 in the US showed that approximately 25% of long-term (≥5 years postdiagnosis) older cancer survivors (aged ≥65 years) developed an SPC. Approximately 25% of smoking-related SPCs were diagnosed among cancer survivors with smoking-related primary cancers. Lung cancer accounted for 31% to 33% of the total mortality from SPCs.^6^

The complex association between cancer survivorship and the risk of SPC underscores the challenges and opportunities for preventive care, including cancer screening in this population. Lung cancer screening (LCS) has been recommended in the US since 2013,^7^ and it is currently recommended in individuals aged 50 to 80 years who have a greater than or equal to 20-pack-year smoking history or, if formerly smoked, they quit within 15 years.^8^ No study has demonstrated a survival benefit of LCS in cancer survivors primarily because the enrollment of cancer survivors in LCS trials has been heterogeneous. For example, the National Lung Screening Trial (NLST)^9^ excluded patients with a history of prostate cancer, whereas the Dutch-Belgian LCS trial excluded patients with a history of breast cancer, renal cancer, and melanoma.^10^

Several organizations recommend extending LCS to high-risk individuals outside the NLST inclusion criterion, including long-term cancer survivors, after 4 years of surveillance without recurrence.^11,12^ This recommendation has not been widely accepted because there are limited data about LCS outcomes in this population. Only 4.1% of NLST participants were cancer survivors.^9^ This study aimed to compare demographic characteristics, LCS LDCT interpretation, and lung cancer detection rate (CDR) in cancer survivors vs those without prior cancer in the general population undergoing LCS.

## Methods

The University of North Carolina at Chapel Hill institutional review board approved this study with a waiver of informed consent, because the study demonstrated no more than minimal risk to participants or their privacy. Reported data were deidentified in accordance with 45 CFR §46.^13^ The cohort study was designed and reported in accordance with the Strengthening the Reporting of Observational Studies in Epidemiology (STROBE) reporting guidelines to ensure transparency, methodological rigor, and comprehensive reporting. We hypothesized that, compared with those with no history of cancer, individuals with a history of cancer would be more likely to have a positive LCS examination and more likely to have a lung cancer diagnosis.

### Data Sources

We used data from the North Carolina Lung Screening Registry (NCLSR), a National Cancer Institute–funded registry that collects prospective data, including demographic characteristics (age, sex, race [categorized as Black, White, and other, which includes American Indian and Alaska Native, Asian and Other Pacific Islander, and any race not otherwise specified], and ethnicity from electronic health record databases), lung cancer risk factors, LCS examination information, follow-up procedures, and outcomes of individuals undergoing LCS at participating imaging sites across North Carolina. We included data on race and ethnicity in this study because there are known differences in lung cancer risk, lung cancer rates, and lung cancer screening rates, completion, and follow-up among racial and ethnic groups. Details of the LCS-eligible and screened population have been previously described.^14^

### Ascertainment of Cancer Survivor Status

Using data from the NCLSR from patients screened for lung cancer at 8 sites in North Carolina from 2015 to 2019, we linked to the North Carolina Central Cancer Registry (NCCCR) data from 2000 to 2020 to ascertain whether a patient had any cancer diagnosis before the baseline LDCT examination and a lung cancer diagnosis after LCS. From the NCCCR, we used information on the primary site and histology to code the prior cancer type (eg, colon or breast) and the diagnosis dates. Where a prior cancer was found, we computed the time between the cancer diagnosis and the LCS. If any individual had more than 1 cancer diagnosis, we used the most recent cancer diagnosis date. We excluded individuals with a previous diagnosis of nonmelanoma skin cancer.

### Outcomes

The LCS examination result was based on the radiologist’s reported LDCT interpretation, using the American College of Radiology’s Lung Imaging Reporting and Data System (Lung-RADS) assessment.^15^ We categorized Lung-RADS into negative (Lung-RADS 1 or 2) or positive (Lung-RADS 3, 4A, 4B, or 4X). We used the NCCCR and NCLSR pathology data to determine whether lung cancer was diagnosed within 1 year of the first LCS examination found in the data. We linked the NCSLR to North Carolina state vital statistics data from 1994 to 2021 to ascertain deaths among the cohort. Data use agreements require the suppression of cell sizes less than 16 in results, and this requirement did not allow us to evaluate lung cancer histology or lung cancer–specific mortality. All patients were monitored for 1 year to ascertain whether lung cancer was diagnosed within 1 year after the first LCS examination.

### Measures

Sociodemographic and clinical characteristics were collected in the NCLSR. For cancer survivors and those without prior cancer, we calculated (1) the probability of developing lung cancer within 5 years using the Lung Cancer Risk Assessment Tool (LCRAT) model^16^ and (2) the probability of dying from lung cancer within 5 years if not undergoing screening using the Lung Cancer Death Risk Assessment Tool (LCDRAT) model through publicly available macros.^17,18,19^ The macros require nonmissing values for the following core variables: age, sex, smoking years and cigarettes per day, and years since quitting smoking (for individuals who formerly smoked). They allow the imputation of missing values for other covariates, including the number of patients with lung cancer, body mass index, education, year of assessment, and the following comorbidity indicators: lung disease, history of cancer, hypertension, coronary heart disease, angina pectoris, heart attack, other heart diseases, stroke, diabetes, bronchitis, kidney failure, liver condition, and health problems requiring special equipment. Six hundred forty-eight of 814 cancer survivors and 4880 of 6481 individuals without a prior cancer had nonmissing core variables. The imputation models were developed using the National Health Interview Survey.^20^

### Statistical Analysis

Data analysis was performed from June 2024 to April 2025. Using χ^2^ tests, we first compared demographic characteristics, including smoking behavior and comorbid conditions at the time of the baseline LDCT examination, between cancer survivors and those without prior cancer. Among cancer survivors, we used coded data from the NCCCR to ascertain the preceding cancer characteristics by sex and the median (IQR) time in years since the prior cancer diagnosis. We then examined the association between previous cancer and the first LCS result, based on the Lung-RADS assessment^15^ of negative or positive, and the lung CDR within 1 year of the first LDCT. We modeled the probability of a positive LDCT result, lung CDR, and all-cause mortality as a function of age, race, sex, and smoking status using logistic regression. Adjusted rates were derived using a logistic regression with the following control variables: age, sex, race, and smoking status. We considered *P* < .05 evidence of a difference or an association; we considered *P* ≥ .05 insufficient evidence of a difference or an association.

## Results

Among our cohort of 7295 individuals screened for lung cancer (mean [SD] age, 64.71 [6.34] years), 814 (11.2%) were cancer survivors (425 men [52.2%]), and 6481 (88.8%) had no prior cancer (3290 men [50.8%]) (Figure). Compared with screened individuals without prior cancer, screened cancer survivors were more likely to be aged 65 years or older vs younger than 65 years (3267 individuals without prior cancer [50.4%] vs 501 cancer survivors [61.6%] aged ≥65 years; χ^2^_1_ = 35.93; *P* < .001), were more likely to be Black than White or other races (871 individuals without prior cancer [13.4%] vs 137 cancer survivors [16.8%] were Black; χ^2^_2_ = 12.46; *P* = .002), and to have formerly vs currently smoked (2728 individuals without prior cancer [42.7%] vs 418 cancer survivors [51.9%] formerly smoked; χ^2^_2_ = 24.62; *P* < .001) (Table 1). Although the rates of diabetes and pneumonia were similar between the 2 groups, cancer survivors had slightly higher rates of respiratory comorbidities, including emphysema and chronic obstructive pulmonary disease, than those without prior cancer (1834 individuals without prior cancer [35.0%] vs 268 cancer survivors [39.9%]; χ^2^_1_ = 6.19; *P* = .01). Cancer survivors also had higher rates of cardiovascular comorbidities, including heart attack or heart disease, hypertension, and stroke, than those without prior cancer (3043 individuals without prior cancer [58.1%] vs 432 cancer survivors [64.4%]; χ^2^_1_ = 9.70; *P* = .002). In the subset of cancer survivors and those without prior cancer who had nonmissing values for the 6 required variables in the LCRAT and LCDRAT models, the median (IQR) 5-year probability of developing lung cancer was similar among the 2 groups (33.0 [17.3-56.5] cases per 1000 in cancer survivors vs 30.2 [16.8-54.2] cases per 1000 in those without prior cancer). Similarly, there was insufficient evidence of a statistically significant difference in the median (IQR) 5-year probability of dying from lung cancer if not screened (22.0 [11.2-40.2] cases per 1000 in cancer survivors vs 19.5 [10.2-36.7] cases per 1000 in those without prior cancer).

**Figure.**
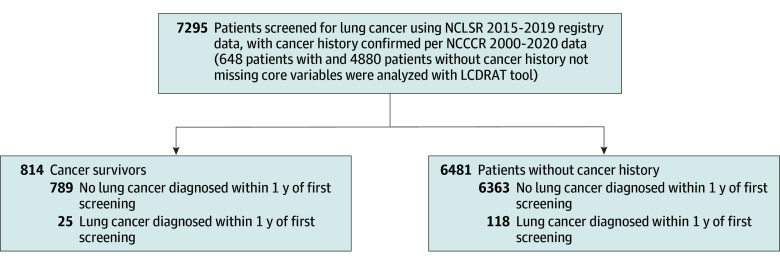
Study Population, North Carolina Lung Screening Registry (NCLSR), 2015 to 2019 LCDRAT indicates Lung Cancer Death Risk Assessment Tool; NCCCR, North Carolina Central Cancer Registry.

**Table 1.** Cohort Characteristics at the Time of Lung Cancer Screening Examination

Characteristic	Participants, No. (%)^a^	
	Cancer survivors (n = 814)	Individuals without prior cancer (n = 6481)
Age, y		
<65	313 (38.5)	3214 (49.6)
≥65	501 (61.6)	3267 (50.4)
Sex		
Female	389 (47.8)	3191 (49.2)
Male	425 (52.2)	3290 (50.8)
Race		
Black	137 (16.8)	871 (13.4)
White	647 (79.5)	5227 (80.7)
Other^b^	30 (3.7)	383 (5.9)
Smoking status		
Current	385 (47.8)	3516 (56.6)
Former	418 (51.9)	2728 (42.7)
Never	<16	41 (0.6)
Missing^c^	<16	196
Smoking pack-years, median (IQR)	40 (25-52)	40 (30-51)
Time since quit, median (IQR), y	4.7 (0.0-10.5)	3.8 (0.0-9.1)
Comorbidities		
Diabetes	142 (21.2)	1120 (21.4)
Pneumonia	41 (5.0)	255 (3.9)
Respiratory^d^	268 (39.9)	1834 (35.0)
Cardiovascular^e^	432 (64.4)	3043 (58.1)
Lung Cancer Risk Assessment Tool score per 1000		
No. of participants	648	4880
Median (IQR)	33.0 (17.3-56.5)	30.2 (16.8-54.2)
Lung Cancer Death Risk Assessment Tool score per 1000		
No. of participants	648	4880
Median (IQR)	22.0 (11.2-40.2)	19.5 (10.2-36.7)

^a^ Cell sizes less than 16 were suppressed because of data use agreement requirements.

^b^ Other included American Indian and Alaska Native, Asian and Other Pacific Islander, and any race not otherwise specified.

^c^ Missing smoking status.

^d^ Respiratory includes chronic obstructive pulmonary disease and emphysema.

^e^ Cardiovascular includes heart disease or heart attack, hypertension, and stroke.

The most common prior cancers among men were prostate cancer (161 men [37.8%]), bladder cancer (48 men [11.3%]), lung cancer (32 men [7.5%]), colorectal cancer (30 men [7.1%]), and head and neck cancer (HNC) (25 men [5.9%]). Among women, the most common prior cancers were breast cancer (193 women [49.6%]), colorectal cancer (23 women [5.9%]), lung cancer (23 women [5.9%]), and HNC (17 women [4.4%]) (Table 2). The median (IQR) interval between the prior cancer diagnosis and the baseline LDCT LCS examination was 5.0 (2.2-8.5) years among men and 5.6 (2.6-9.5) years among women. The time since prior cancer diagnosis was less than 5 years for 211 men (49.7%) and 174 women (44.7%).

**Table 2.** Characteristics of Prior Cancer Among Cancer Survivors, by Sex

Type of prior cancer	Participants, No. (%)^a^	
	Men (n = 425)	Women (n = 389)
Bladder	48 (11.3)	<16
Brain or other central nervous system	<16	17 (4.4)
Breast	<16	193 (49.6)
Cervical	NA	<16
Colorectal	30 (7.1)	23 (5.9)
Endocrine	<16	17 (4.4)
Esophagus	<16	0
Kidney	21 (4.9)	<16
Head and neck^b^	25 (5.9)	17 (4.4)
Leukemia	<16	<16
Lung	32 (7.5)	23 (5.9)
Melanoma	29 (6.8)	19 (4.9)
Non-Hodgkin lymphoma	<16	<16
Prostate	161 (37.8)	NA
Uterus	NA	<16
Other^c^	55 (12.9)	74 (19.0)
Time since prior cancer diagnosis, y		
Median (IQR)	5.0 (2.2-8.5)	5.6 (2.6-9.5)
Category		
<5	211 (49.7)	174 (44.7)
5 to <10	146 (34.4)	134 (34.5)
>10	68 (16.0)	81 (20.8)

Abbreviation: NA, not applicable.

^a^ Cell sizes less than 16 were suppressed because of data use agreement requirements.

^b^ Head and neck cancer includes oral cavity and laryngeal cancers.

^c^ Other includes Hodgkin disease, liver, multiple myeloma, ovary (women), soft tissue, or stomach cancers.

The proportion of positive LDCT results (Lung-RADS 3, 4A, 4B, or 4X) was 15.8% (120 of 758 cancer survivors) among cancer survivors and 17.0% (1032 of 6059 individuals) among those without prior cancer (χ^2^_1_ = 0.86; *P* = .35). The unadjusted odds ratio (OR) for having a positive LCS result was 0.93 (95% CI, 0.76-1.15). After adjustment for age, sex, race, and smoking status, the likelihood of having a positive LCS result was similar (adjusted OR, 0.91; 95% CI, 0.74-1.12) (Table 3).

**Table 3.** Comparison of Lung-RADS in Cancer Survivors vs Those Without Prior Cancer, 2014-2019

Lung-RADS categorization and assessment (probability of malignancy)	Participants, No. (%)		OR (95% CI)	
	Cancer survivors (n = 758)^a^	Individuals without prior cancer (n = 6059)^a^	Unadjusted^b^	Adjusted^b^^,^^c^
Negative			0.93 (0.76-1.15)	0.91 (0.74-1.12)
1, Negative (<1%)	283 (37.3)	2505 (41.3)		
2, Benign (<1%)	355 (46.8)	2514 (41.5)		
Positive				
3, Probably benign (1%-2%)	55 (7.3)	595 (9.8)		
4A, Suspicious (5%-15%)	40 (5.3)	267 (4.4)		
4B or 4X, Suspicious (>15%)	25 (3.3)	170 (2.8)		

Abbreviations: Lung-RADS, American College of Radiology Lung Imaging Reporting and Data Assessment; OR, odds ratio.

^a^ Individuals with missing or unknown Lung-RADS were excluded from this analysis.

^b^ Adjusted and unadjusted ORs are shown for having a positive low-dose computed tomography (Lung-RADS 3, 4A, 4B, or 4X).

^c^ ORs and 95% CIs were adjusted for age, sex, race, and smoking status.

In the 1 year following the first LCS examination, there were 25 lung cancers diagnosed among cancer survivors and 118 diagnosed among those with no prior cancer. The unadjusted CDR was 30.4 cases per 1000 among cancer survivors vs 18.2 per 1000 among those without prior cancer (OR, 1.69; 95% CI, 1.08-2.62; *P* = .02). The adjusted lung CDR was 26.0 cases per 1000 (95% CI, 17.0-38.2 cases per 1000) among cancer survivors vs 17.0 cases per 1000 (95% CI, 14.1-20.6 cases per 1000) among those without prior cancer (adjusted OR, 1.51; 95% CI, 0.97-2.36; χ^2^_1_ = 3.38; *P* = .07). During the 1 year following the baseline LCS examination, there were 16 deaths among cancer survivors and 114 deaths among those without prior cancer. There was insufficient evidence of a statistically significant difference in the adjusted mortality rate per 1000 between cancer survivors and those with no history of cancer (19.4 cases per 1000 [95% CI, 12.0-31.3 cases per 1000] vs 17.1 cases per 1000 [95% CI, 14.1-20.6 cases per 1000]; χ^2^_2_ = 0.25; *P* = .62) (Table 4).

**Table 4.** Comparison of Lung Cancer Detection Rate and All-Cause Mortality Rates in Cancer Survivors vs Those Without Prior Cancer, 2014-2019

Outcome	Participants No. (%)		*P* value^a^	OR (95% CI)	
	Cancer survivors (n = 814)	Individuals without prior cancer (n = 6481)		Unadjusted	Adjusted^b^
Lung cancer diagnosed within 1 y of LCS					
Yes	25 (3.1)	118 (1.8)	NA	1.69 (1.08-2.62)	1.51 (0.97-2.36)
No	789 (96.9)	6363 (98.2)			
Adjusted lung cancer detection rate, cases per 1000 (95% CI)^b^	26.0 (17.0-38.2)	17.0 (14.1-20.6)	.07	NA	NA
All cause death within 1 y of LCS					
Yes	16 (2.0)	114 (1.8)	NA	1.15 (0.68-1.94)	1.14 (0.68-1.92)
No	798 (98.0)	6367 (98.2)			
Adjusted all-cause mortality rate, cases per 1000 (95% CI)^b^	19.4 (12.0-31.3)	17.1 (14.1-20.6)	.62	NA	NA

Abbreviations: LCS, lung cancer screening; OR, odds ratio.

^a^ *P* value comparing Lung Imaging Reporting and Data Assessment (Lung-RADS) 1 or 2 with Lung-RADS 3, 4A, 4B, or 4X.

^b^ Lung cancer detection rate, all-cause mortality rate, and OR are adjusted for age, sex, race, and smoking status.

## Discussion

In this cohort study, cancer survivors who underwent LCS were older, were more likely to have formerly smoked, and had higher rates of respiratory and cardiovascular comorbidities compared with LCS-screened individuals without prior cancer. Yet, we found that cancer survivors and those without a history of cancer had similar rates of positive LDCT findings, comparable rates of the median 5-year probability of developing lung cancer, and similar mortality rates at 1 year after LCS. Both the median 5-year score of dying from lung cancer if not screened and the lung CDR were slightly higher among cancer survivors than those with no history of cancer, although there was insufficient evidence of a statistical difference.

Our findings align with our secondary analysis of 1071 cancer survivors (4.1%) participating in the NLST.^21^ We observed that cancer survivors were more likely to be older than 65 years and had higher rates of respiratory comorbidities (chronic obstructive pulmonary disease and emphysema) and hypertension compared with those without prior cancer. In addition, the 2 groups had similar age-adjusted rates of positive LDCT findings at baseline LDCT. The age-adjusted lung CDR per 100 in the NLST was higher among cancer survivors vs those without prior cancer (1.9% vs 0.8%; relative risk, 2.51; 95% CI, 1.67-3.81).^21^ The median time from prior cancer to LCS was 9 years for men and 10 years for women. Approximately 23.9% of men and 8.3% of women underwent a baseline LDCT within 5 years of the prior cancer diagnosis.^21^ A recent single-institution study of 543 cancer survivors (38% with breast, 19% with HNC, and 16% with lung cancer) with a mean age of 66 years who underwent LCS found a rate of a positive LDCT at baseline of 22%, higher than the 15.8% rate found in our study. Similar to our findings, the median interval between prior cancer diagnosis and inclusion in LCS was 6 years.^22^

Our findings that breast, prostate, bladder, lung, and colorectal cancer and HNC were the most common prior cancers among cancer survivors corroborate with existing data, including international data.^5,22,23,24^ In a large Danish cohort study, a higher cumulative incidence of an SPC was noted among individuals with prior HNC and prior bladder cancer.^5^ In a cohort of 2 116 163 cancer survivors from Surveillance, Epidemiology, and End Results data from 1992 to 2008, patients with a history of bladder cancer had the highest risk of developing a second cancer.^23^ A meta-analysis of 15 studies, including 1 161 979 patients, showed that women with a history of breast cancer had a much higher risk of developing a second primary lung cancer (SPLC), with an incidence ratio of 1.25 (95% CI, 1.12-1.39; *P* < .001). Furthermore, smoking (OR, 9.73; 95% CI, 6.86-13.82; *P* < .001) and prior radiotherapy (relative risk, 1.40; 95% CI, 1.19-1.66; *P* < .001) were associated with increased risk of developing an SPLC.^24^

The complex association between cancer survivorship and the risk of an SPC underscores both the challenges and the opportunities for delivering preventive care, such as screening for lung cancer, the most common SPC, among the rapidly growing population of cancer survivors. This population represents a clinically heterogeneous population characterized by differences in underlying comorbidities, including long-term adverse effects from prior cancer diagnosis and treatments. Cancer treatment can accelerate aging,^25,26^ leading to an accumulation of chronic conditions, physical disabilities, frailty, and cognitive impairment in cancer survivors. Expanding LCS to eligible cancer survivors in the population may be challenging because of concerns about a higher prevalence of comorbid conditions and an increased risk of more positive LDCT findings requiring follow-up. These factors can impact the net benefits of LCS and its role in improving survival outcomes. Despite these complexities, a history of cancer does not negatively affect the survival of an SPLC.^27^

In contrast to prior findings of higher rates of positive LDCT at the first LCS examination, potentially attributable to changes in lung parenchyma (eg, inflammation or fibrosis) caused by prior chemotherapy or radiation therapy among cancer survivors,^22^ we did not observe such a difference. Further research on the outcomes of LCS among cancer survivors is needed to evaluate the net benefit of LCS in this growing population. Moreover, research on improved imaging protocols and artificial intelligence-assisted interpretations may help enhance the diagnostic accuracy of LDCT in cancer survivors.

### Strengths and Limitations

To our knowledge, this is the largest study to date comparing LCS LDCT results, lung CDR, and all-cause mortality among cancer survivors and individuals without a history of cancer in the general population undergoing LCS. Our study is strengthened by the multisite representation of LCS in the population and the linkage with state cancer registry data to confirm prior cancer diagnoses. Our study also has several limitations. First, although the NCSLR data are collected from multiple sites, including academic and community health care settings, they are from 1 state, and differences in cancer survivors LCS across the US may exist. Second, the small cancer survivors sample size may limit the precision of the estimates. Third, there is also potential misclassification of personal cancer history (eg, individuals who moved into the state after receiving a cancer diagnosis). Fourth, lung cancer mortality could not be evaluated because of the small case numbers and restrictions on sharing cell counts fewer than 16. Fifth, missing values for core variables required for the LCRAT and LCDRAT macros may have engendered bias for risk score analyses. Sixth, among the subset of cancer survivors with prior lung cancer, we were unable to confirm that the screen-detected lung cancer was a second primary lung cancer vs a recurrence of the primary lung cancer.

## Conclusions

In this study, rates of LDCT were similar between cancer survivors and individuals without prior cancer. Prioritizing LCS in cancer survivors to assess effectiveness and outcomes is an area of potential high impact. For one, we expect a substantial increase in the number of cancer survivors in the next 2 decades. Moreover, this population is at increased risk of developing an SPLC. Although age and smoking history are a practical way to select individuals at risk for lung cancer who may benefit from LCS, consideration of clinical variables associated with increased risk of lung cancer, such as a history of cancer, should be evaluated in the context of individualized shared decision-making and assessment of net benefits and risk of LCS. Further research in this growing population may help refine screening protocols and enhance survivorship care.

## Notes

Supplement. Data Sharing Statement
