# Supplementary material for: Lung Cancer Screening in Cancer Survivors vs Those Without a History of Cancer
Source: JAMA Netw Open. 2025 Sep 30;8(9):e2535000. doi: 10.1001/jamanetworkopen.2025.35000 (PMC12485636; doi:10.1001/jamanetworkopen.2025.35000)
Supplement: Supplement. — Data Sharing Statement [file jamanetwopen-e2535000-s001.pdf]

Data Sharing Statement

Rivera. Lung Cancer Screening in Cancer Survivors vs Those Without a History of Cancer. *JAMA Netw Open*. Published October 02, 2025. doi:10.1001/jamanetworkopen.2025.35000

Data
Data available: No

Additional Information
Explanation for why data not available: The data for this study are obtained from data linked across several sources, including data exports from health systems, electronic health records, cancer registry records, and vital status records. The use of this data is restricted by established data use agreements and is restricted in terms of being shared.
